# Influence of femoral external shape on internal architecture and fracture risk

**DOI:** 10.1007/s10237-019-01233-2

**Published:** 2019-11-08

**Authors:** C. C. Villette, J. Zhang, A. T. M. Phillips

**Affiliations:** 1 The Royal British Legion Centre for Blast Injury Studies, Imperial College London, London, UK; 2 Structural Biomechanics, Department of Civil and Environment Engineering, Imperial College London, London, UK; 3 Auckland Bioengineering Institute, University of Auckland, Auckland, New Zealand

**Keywords:** Femur morphology, Internal architecture, Statistical shape model, Fracture risk, Structural finite element model, Computational efficiency

## Abstract

The internal architecture of the femur and its fracture behaviour vary greatly between subjects. Femoral architecture and subsequent fracture risk are strongly influenced by load distribution during physical activities of daily living. The objective of this work is to evaluate the impact of outer cortical surface shape as a key affector of load distribution driving femoral structure and fracture behaviour. Different femur cortical shapes are generated using a statistical shape model. Their mesoscale internal architecture is predicted for the same activity regime using a structural optimisation approach previously reported by the authors and fracture under longitudinal compression is simulated. The resulting total volume of bone is similar in all geometries although substantial differences are observed in distribution between trabecular and cortical tissue. Greater neck-shaft and anteversion angles show a protective effect in longitudinal compression while a thinner shaft increases fracture risk.

## Introduction

A significant inter-subject variability in femoral fracture patterns is commonly observed. Proximal femur fractures reported in the literature for longitudinal compression or side fall range from subcapital to subtrochanteric including intertrochanteric, and from simple lines to multiple fragments (Cristofolini et al. 2007; Keyak et al. 1997; de Bakker et al. 2009). The boundary conditions constraining the patient fall or the in-vitro tests are influential (Yang et al. 1996; Cumming and Klineberg 1994; Villette 2016), but it has also been suggested that specific bone shapes (or morphologies) might constitute predispositions to fracture and potentially influence the fracture type (Gregory and Aspden 2008; Bryan et al. 2009; Whitmarsh et al. 2011). For instance, a longer hip axis has been correlated with an increased fracture risk (Boonen et al. 1995; Gnudi et al. 1999; Nakamura et al. 1994). So has a larger neck-shaft angle (Whitmarsh et al. 2011; Alonso et al. 2000) although other studies disagree (Kukla et al. 2002). The aim of this study was to investigate the effects of femur shape on fracture behaviour. It has been established that bone presents the ability to adapt towards an internal architecture optimised to the loading conditions experienced (Wolff 1869; Frost 1987; von Meyer 1867). This characteristic of bone tissue implies that the bone outer shape, which impacts the loading distribution internally, has an influence on inner structure, and thus on structural failure behaviour. In this study, seven femoral geometries with quantified morphological variations were built using a statistical shape model (Zhang et al. 2014, 2016) and used to generate as many femur structural models optimised to the same set of loading conditions, following the strain-based structural optimisation framework described in Phillips (2012); Phillips et al. (2015) and Villette and Phillips (2017). These femurs were then taken to fracture in a compressive longitudinal loading scenario using a damage elasticity model previously released by the authors (Villette and Phillips 2018). Trends in correlation between morphometrics and fracture patterns were then investigated.

## Methods

### Overview

**Fig. 1:**
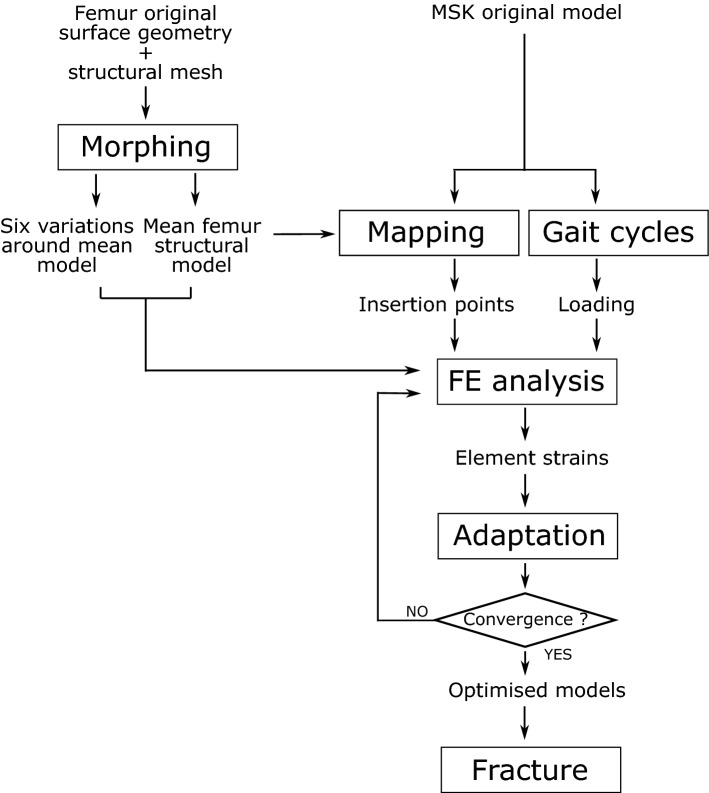
Framework adopted to assess the influence of femur morphology on internal structure and fracture behaviour

Figure 1 illustrates the framework adopted in this study. An initial structural femur mesh made of beam and shell finite elements (FE) was obtained using the process described in Phillips et al. (2015), based on the outer geometry extracted from a CT scan. This mesh was then morphed using a principal components analysis (Zhang et al. 2014, 2016) to produce a structural mesh with a surface geometry representative of a mean femur across a typical western urban population. Six other meshes were also generated whose outer geometries differed from the mean geometry by plus/minus two standard deviations along the first four principal components. The shape differences included neck-shaft angle, shaft width, and anteversion angle. Muscle insertion points from a musculoskeletal (MSK) model validated at the hip (Modenese et al. 2011, 2013; Modenese and Phillips 2012a) were mapped onto these geometries, using host-mesh fitting (Zhang et al. 2014; Fernandez and Hunter 2005). The structural strain-based optimisation described in Phillips et al. (2015) was then performed on each femur to predict their internal architecture based on the loading conditions associated with the most frequent activities of daily living including walking, stair ascent and descent, sit-to-stand and stand-to-sit (Morlock et al. 2001). These loading conditions were kept identical for all femurs and applied on the mapped points of muscle insertion. The biofidelic femur models obtained were then submitted to the quasi-static longitudinal compression fracture scenario described in Villette and Phillips (2018). Differences in inner structure and fracture responses between the femurs were assessed.

### Generation of parametric femur geometries

#### Statistical femur shape model

**Fig. 2:**
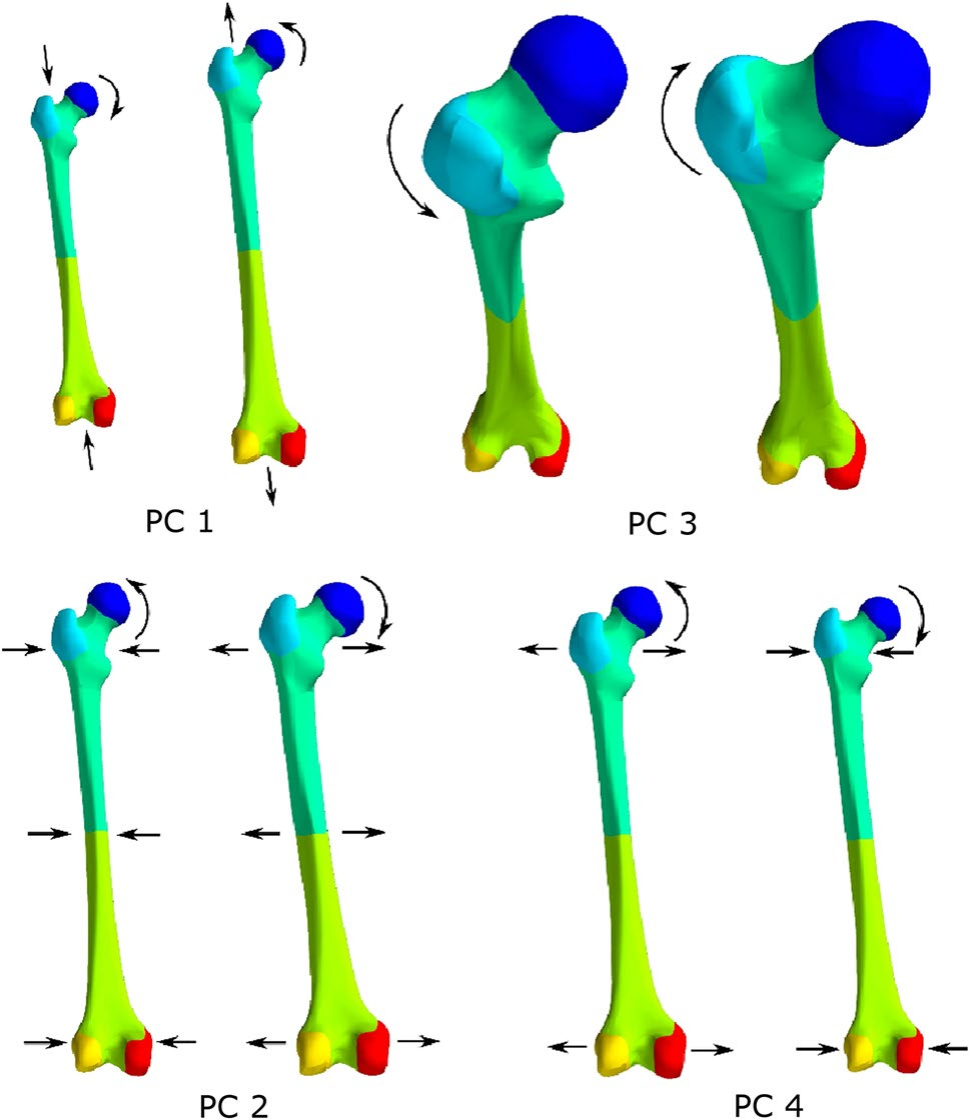
Shape variations associated with the first four principal components of the whole femur statistical shape model Adapted from Zhang et al. (2014)

Zhang et al. (2014, 2016) developed an anatomical statistical shape model of the femur. It involved the isolation of a set of independent principal components capable of capturing over 95$\%$ of variation in femoral geometry across a typical western urban population, with donor age ranging from 19 to 95 years old and composed of 110 males and 94 females. Briefly, a training set of manually segmented femur surfaces were partitioned according to Gaussian curvature. Mean-shift clustering was used to identify the most stable regions describing the femur surfaces. Reference piecewise parametric meshes were fitted to each region and used to train regional statistical shape models through fitting training iterations. Fitted region meshes were then assembled into full femur meshes for training a whole femur model. The morphing process involved in the generation of this statistical shape model also allows for automatic computation of some characteristic morphometric measurements such as the femoral neck axis length or the neck-shaft angle. The initial model developed by Zhang et al. (2014) was based on the outer geometry of 41 femurs. A refined version of the model was then generated, based on 204 femurs, which also accounted for cortical thickness (Zhang et al. 2016). The version used in this study corresponds to the first model (no consideration of cortical thickness) modified to include all 204 femurs. Four principal components (PCs) were sufficient to account for over 98$\%$ of the total femoral morphology variation across the population. The key morphological changes associated with these PCs are described in Fig. 2. The first PC, accounting mainly for the variation in overall length, accounted for $91\%$ of the total variation on its own. The next three components can be loosely interpreted as evaluations of neck-shaft angle combined with shaft width (PC 2), neck anteversion angle (PC 3), and neck-shaft angle (PC 4). After normalisation for size, they accounted for 40$\%$, 22$\%$, 18$\%$ of total variation, respectively.

#### Mesh generation

An initial structural femur mesh was obtained using the process described in Phillips et al. (2015), based on the outer geometry extracted from a CT scan (male subject, 78 kg, 175 cm, 27 years old) provided by the Royal British Legion Center for Blast Injury Studies at Imperial College London. This structural mesh is made of a lattice of truss elements to represent trabecular bone contained within a layer of shell elements representing the cortex. The initial mesh is randomised, with truss elements of uniform 0.1 mm radius distributed over the entire bone volume with no preferential directionality, and shell elements with uniform 0.1 mm thickness.

**Fig. 3:**
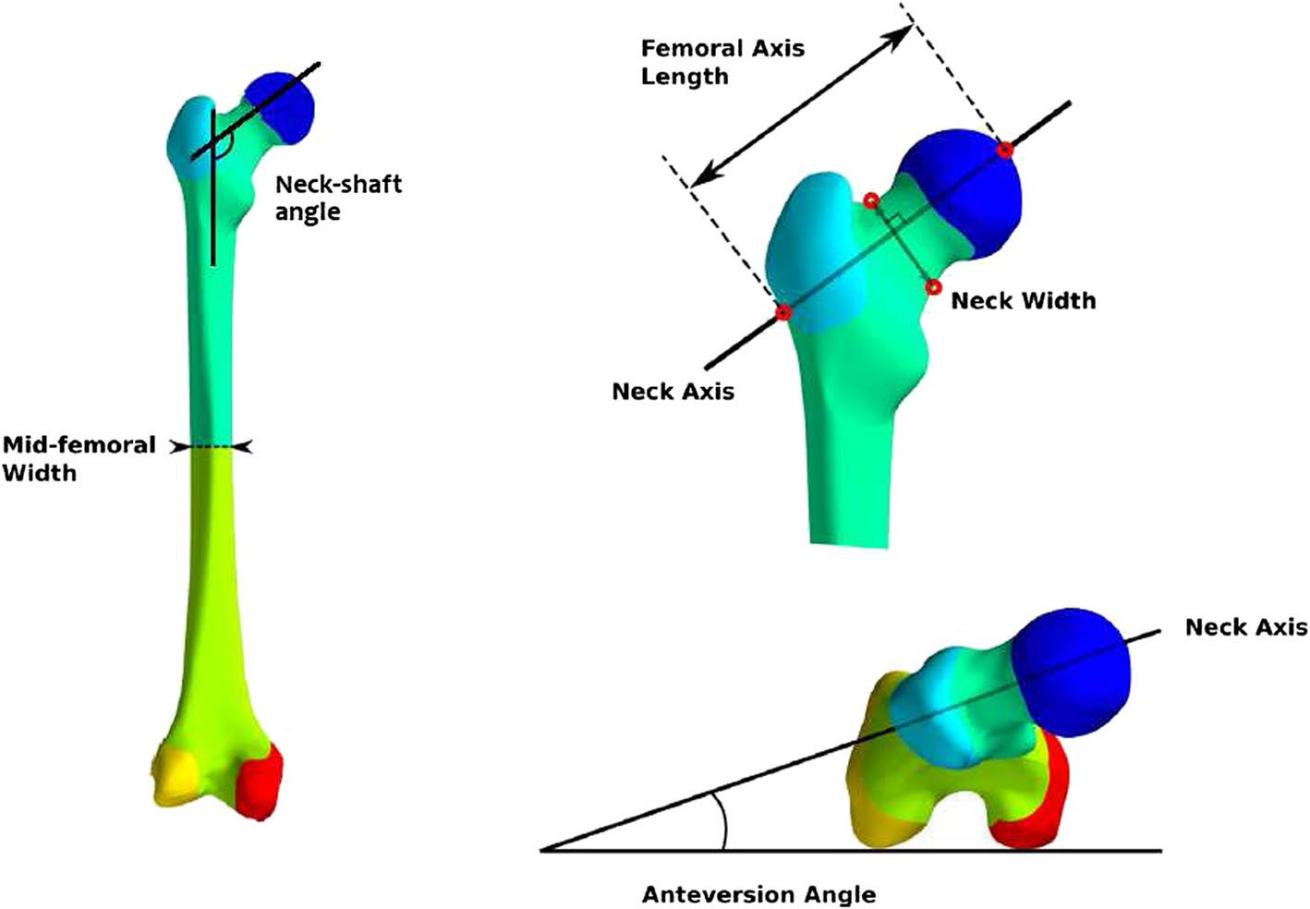
Definition of the characteristic morphometric measurements used in this study. The femoral axis length (FAL) corresponds to the linear distance from the base of the greater trochanter to the apex of the femoral head (Center et al. 1998). The hip axis length (HAL) mentioned in the Discussion is aligned with the FAL and corresponds to the linear distance from the base of the greater trochanter to the inner pelvic rim (Gregory and Aspden 2008; Center et al. 1998) Adapted from Zhang et al. (2016)

The above initial structural mesh was morphed to seven femur geometries generated from the shape model. A femur outer geometry of equal length to that used in the MSK model, representative of the average across the population, was first generated by setting all principal components except for PC 1 to their mean value. Six other geometries were generated by varying PC 2, PC 3 and PC 4 by two standard deviations on both sides of the mean. The corresponding initial structural meshes were generated by morphing the initial structural mesh onto the new geometries using host-mesh fitting. Variations in size (PC 1) were not investigated, which also served to maintain consistency between the FE and MSK models. The outer geometries of the meshes used in this study are described in terms of PCs and characteristic morphometric measurements in Table 1. The definitions of these morphometric measurements are given in Fig. 3.

**Table 1:** Description of the femoral geometries generated

Model	Mean	2a	2b	3a	3b	4a	4b
PC 2	M	M − 2SD	M + 2SD	M	M	M	M
PC 3	M	M	M	M − 2SD	M + 2SD	M	M
PC 4	M	M	M	M	M	M − 2SD	M + 2SD
NS angle ($^{\circ }$)	125	130	121	121	124	132	110
AV angle ($^{\circ }$)	18	29	8	5	28	12	22
FAL (mm)	94	85	104	90	98	97	91
Shaft w. (mm)	26	22	30	26	26	25	27
Neck w. (mm)	36	34	38	34	41	37	37

*M* mean, *SD* standard deviation, *NS* neck-shaft, *AV* anteversion, *FAL* femoral axis length, *w.* width

### MSK simulations and structural optimisation

The initial structural meshes were individually, iteratively adapted towards a structure optimised for the loading experienced during activities of daily living using a strain-based algorithm adapted from Phillips et al. (2015). Briefly, the structural optimisation algorithm relies on Frost’s (1987) *Mechanostat* principle to iteratively reduce or increase the cross section of individual elements based on the difference between their experienced strain and a target strain set to 1250 $\upmu \epsilon$. Cortical shell thicknesses vary between 0.1 and 8 mm and trabecular truss radii between 0.1 and 2 mm.

The loading conditions (muscle forces, joint contact forces and inertial loading) involved in activities of walking, stair ascent, stair descent, sit-to-stand and stand-to-sit were taken from Phillips et al. (2015). In that study, the musculoskeletal model of the lower limb is based on the anatomical dataset published by Klein Horsman et al. (2007) and implemented in OpenSim (Delp et al. 2007). The MSK model fully described in previous work (Phillips et al. 2015; Modenese et al. 2011) includes six segments (pelvis, femur, patella, tibia, hindfoot and midfoot plus phalanxes) connected by five joints (pelvis-ground connection, acetabulofemoral (hip) joint, tibiofemoral (knee) joint, patellofemoral joint and ankle joint). Thirty-eight muscles of the lower extremity are represented through one hundred sixty-three actuators, whose path is enhanced by frictionless via points and wrapping surfaces.

Host-mesh fitting (Zhang et al. 2014; Fernandez and Hunter 2005) was used to morph the muscle insertion points used in Phillips et al. (2015) onto the mesh associated with the mean femur geometry. The corresponding node IDs were kept as the points of muscle load application in all seven models. Finally, all seven models were realigned consistently with the coordinate frame recommended by ISB (Wu et al. 2002), using manual location of bony landmarks.

The volumes of trabecular bone ${V}_{\text {Trabecular}}$, cortical bone ${V}_{\text {Cortical}}$, and the total bone volume ${V}_{\text {Total}}$ in the converged femur models were calculated as:

1 $$\begin{aligned} {V}_{{\text {Trabecular}}}&=\sum _{j}{\pi }r_{j}^2l_{j} \end{aligned}$$

2 $$\begin{aligned} {V}_{\text {Cortical}}&=\sum _{j}A_{j}t_{j} \end{aligned}$$

3 $$\begin{aligned} {V}_{\text {Total}}&={V}_{\text {Trabecular}}+{V}_{\text {Cortical}} \end{aligned}$$

with subscript *j* referring to the $j{\mathrm{th}}$ truss (trabecular bone) or shell (cortical bone) element, *r* the truss radius, *l* the truss length, *A* the area of the shell face and *t* the shell thickness.

### Fracture simulations

The influence of femur outer morphology on fracture risk, including fracture load and fracture pattern, was assessed using the scenario of quasi-static longitudinal loading described in Villette and Phillips (2018) with the damage elasticity model developed as an *Abaqus* subroutine for 75-year-old bone material (Villette and Phillips 2018). In a few words, this scenario models a vertical displacement-driven compression with the femoral shaft oriented at $7^{\circ }$ with respect to the vertical direction in the frontal plane, and with the distal femur fixed. The femoral head is overlayed with four layers of PMMA. The constrained displacement boundary condition is assigned as a ramp to the point at the top PMMA layer located on the vertical axis. The trabecular trusses are replaced with beam elements to account for bending. At each time step, the strains associated with each section point are used to update the Young’s modulus assigned to the corresponding section point following a damage elasto-plastic behaviour defined prior to the simulation and dependant on the age of the bone considered. At the end of the plastic regime, upon reaching the ultimate strain, the Young’s modulus is set three orders of magnitude lower than that of intact bone (${E}_{\text {intact}} = 18$ GPa), which effectively cancels the load bearing ability in that region of the model. Global structural failure is reached when the overall structural response (sum of the vertical reaction forces at the fixed nodes as a function of the prescribed displacement) shows a sudden drop. The failed elements are identified, which allows the visualisation of the fracture pattern. The failure load is taken as the maximum reaction force recorded over the simulation.

## Results

### Structural bone changes

The percentage differences in bone volume between the mean femur and the six others are presented in Table 2. Bone volume amounts to $204.6\,\hbox {cm}^3$ in the mean model, composed of $123.3\,\hbox {cm}^3$ of cortical bone and $81.3\,\hbox {cm}^3$ of trabecular bone. The total bone volume shows little variation between the models, with a maximum difference to the mean femur of 3$\%$, reached by Femur 4b. Variability in distribution is more important, with close to 16$\%$ difference in trabecular bone between Femur 2a and the mean femur.

**Table 2:** Percentage difference in bone volume between the mean femur model and the six others

Model	2a	2b	3a	3b	4a	4b
Cortical	− 11.8	+ 9.4	−5	+ 0.9	− 4	+ 2.2
Trabecular	+ 15.9	− 11.7	+ 12.6	− 0.2	+ 3.4	+ 4.2
Total bone volume	− 0.7	+ 1	+ 2	+ 0.5	− 1.1	+ 3

**Fig. 4:**
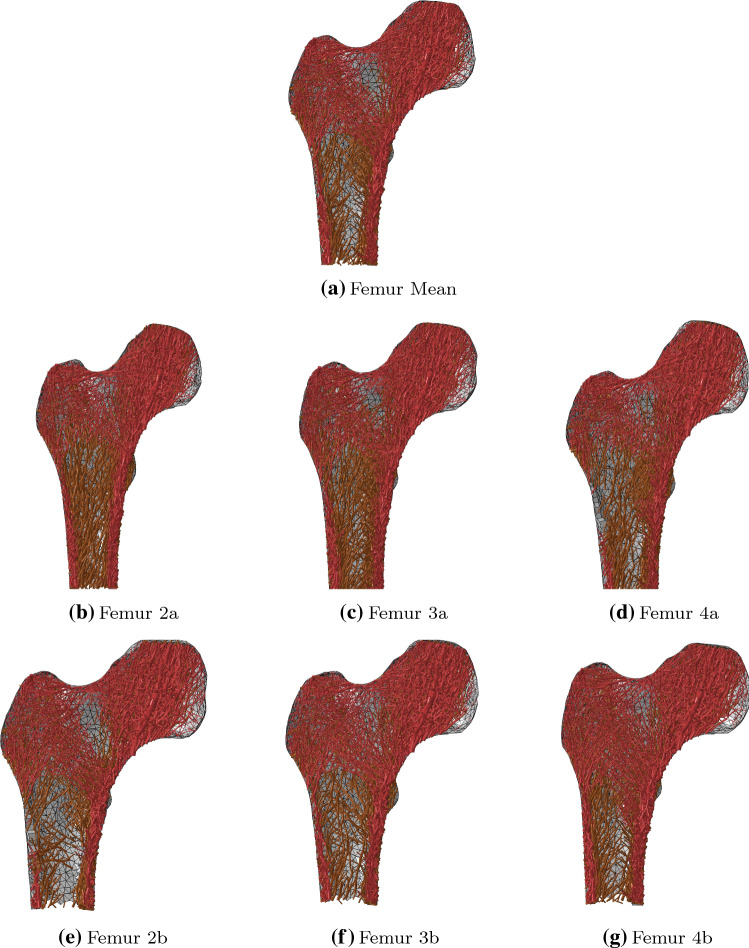
Overlay plots of proximal frontal cuts of the converged femur models. The red front layer displays the central 10 mm slice of trabecular bone elements with a radius $r>$ 0.1 mm. Behind it is a brown layer of all other trabecular elements within the depth of the bone with radius $r>$ 0.3 mm. At the back is the posterior cortex shown in grey

**Fig. 5:**
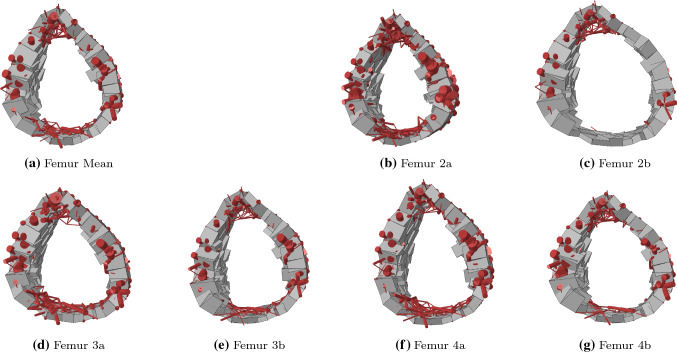
Transverse 10-mm-thick slices at mid-shaft of the converged femur models with trabecular bone shown in red and cortical bone in grey

Figures 4 and 5 present proximal frontal cuts and transverse shaft slices of all seven converged structural femur models. The main trabecular groups described in the literature (von Meyer 1867) can be observed in all models, although spatial density of elements vary.

### Fracture patterns

**Fig. 6:**
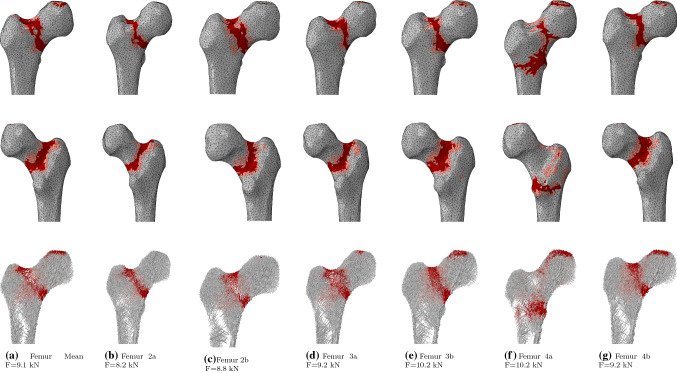
Fracture patterns in anterior (top) and posterior (middle) views of the cortex and frontal cut (bottom) of the trabecular bone. Yielded and failed elements are displayed in pink and red, respectively

The failure loads for all seven models considered as well as their percentage difference to the failure load of the mean femur are displayed in Table 3. The mean femur failed at 9.1 kN. The other femurs presented failure loads within 12$\%$ of that value, with an average of 9.3 kN. All femurs fractured in the neck, except for Femur 4a which presented an intertrochanteric pattern. The fracture patterns are displayed in Fig. 6.

**Table 3:** Failure loads and percentage difference to the mean femur

Model	Mean	2a	2b	3a	3b	4a	4b
Failure load (kN)	9.1	8.2	8.8	9.2	10.2	10.2	9.2
$\%$Difference to mean	–	− 10	− 3	+ 1	+ 12	+ 12	+ 1

## Discussion

### Influence of bone morphology on inner structure and fracture patterns

After convergence of the structural optimisation, negligible difference in total bone volume was observed between the mean femur model and the six others. Substantial differences of up to 16$\%$ were, however, observed in total trabecular or cortical volume. In all cases, a substantial increase in the volume of one type of bone was compensated by a substantial decrease in the volume of the other bone type. This observation is consistent with the concept of structural optimisation, in which a similar amount of bone is differently distributed to adapt to specific deformation modes. A clear example of this phenomenon is given by models 2a and 2b which show a respective difference of + 16$\%$ and − 12$\%$ in trabecular volume compared to the mean femur and a respective difference of − 12$\%$ and + 9$\%$ in cortical volume. Compared to the mean femur, their geometry presents a smaller and larger shaft outer radius, respectively (Table 1). This observation is consistent with the results reported by Zhang et al. (2016), who found that, across a population of over 200 femurs, reduced shaft width was correlated with reduced shaft cortical thickness. This is again consistent with the concept of structural optimisation as increasing the cortical thickness in the shaft is more efficient as a stiffening mechanism if the shaft has a larger diameter. Indeed, bending of the shaft is a primary deformation mode for the femur, particularly for walking or stair ascent activities. In a simple representation of the bone, where the shaft would be considered as a beam with hollow cylindrical cross section in bending, a larger outer radius represents an advantage in resisting bending as the cross-sectional second moment of area *I* is related to the fourth power of the outer $r_{\mathrm{o}}$ and inner $r_{\mathrm{i}}$ radii:

4 $$\begin{aligned} I = \frac{\pi (r_{\mathrm{o}}^4 - r_{\mathrm{i}}^4)}{4} \end{aligned}$$

This hypothesis is further supported by the differences in cross-sectional geometry of the shaft in the mean femur, Femur 2a and Femur 2b, presented in Fig. 5a, b, c. The reduced volume of cortical bone observed for Femur 3a, whose geometry presents a smaller anteversion angle, could originate from the deformation mode switching from the biaxial bending observed for the mean femur to a deformation profile closer to uniaxial bending in the frontal plane, which requires less stiffening of the cortex on the anterior and posterior sides. Interestingly, Heller et al. (2001) reported the observation of higher hip contact forces (measured on instrumented hips) for a femur with a greater anteversion angle, which is also consistent with increased cortex volume being associated with larger anteversion angles. Finally, the slight decrease and increase in cortical bone volume observed for Femur 4a and Femur 4b, respectively, might originate from the changes in moment arm of the hip contact force, which increases as the neck-shaft angle decreases, leading to a greater bending deformation. The observed increase in cortical volume with decreasing neck-shaft angle is also consistent with the observations reported by Zhang et al. (2016).

The failure loads observed for Femurs 4a and 4b suggest that a greater neck-shaft angle is associated with a decreased fracture risk in a vertically loaded simulation. Most clinical studies described in the literature reported the opposite trend (Gregory and Aspden 2008; Whitmarsh et al. 2011; Gnudi et al. 2002; Partanen et al. 2001). In addition, several studies have reported the association of a greater neck-shaft angle with cervical fracture rather than intertrochanteric (Gnudi et al. 2002; Partanen et al. 2001), which is in opposition with the patterns observed in this study, where the only intertrochanteric fracture obtained was associated with the greatest neck-shaft angle tested. These results are, however, in agreement if they are analysed in terms of the bending moment generated in the bone. Indeed, the great majority of clinical studies are based on side fall data. In such a scenario, the moment generated at the fixed point in the trochanter increases with the neck-shaft angle. In all the studies cited here, the increased fracture risk is thus associated with an increased internal moment, and cervical fractures are favoured over intertrochanteric when this internal moment is higher. It is also worth considering the effects of cortical adaptation to a higher neck-shaft angle; as illustrated with Femurs 4a and 4b, a thinner cortex is sufficient for a femur with higher neck-shaft angle to support a longitudinal load because the associated bending moment is lower. However, the thinner cortex makes the bone more prone to failure in side-impact. This study models fracture under longitudinal compressive loading with the distal femur fixed. In this case, a higher neck-shaft angle generates a smaller internal moment. Just as in the clinical studies, the present results indicate a higher fracture risk associated with a greater internal moment, and an increased tendency towards intertrochanteric fractures when that moment is lower. It is interesting to note that studies report a greater neck-shaft angle in the active population (Anderson and Trinkhaus 1998), more likely to generate high contact forces at their joints, and thus high internal moments in their femur. Most bipedal activities are likely to load the bone in a similar fashion as here, with a high downwards vertical force on the femoral head. The greater neck-shaft angle of the active population thus constitutes a beneficial protection against the high internal moments they are likely to generate.

Based on the failure loads obtained for Femurs 3a and 3b, it can be inferred that a higher anteversion angle has a protective effect on the femur. It is interesting to note that the fracture load obtained for Femur 3b is 12 $\%$ larger than that of the mean femur while Femur 3b is the closest of the six models to the mean femur in terms of relative amount of cortical and trabecular bone (Table 2). Little is reported in the literature regarding the potential influence of anteversion angle on femoral fracture, although this feature becomes of importance when orientating a hip implant (Tayton 2007; Heller et al. 2001). A possible cause for this rare investigation might be the two-dimensional nature of the data often used by clinicians, typically X-ray taken in the frontal plane, where anteversion angle cannot be computed. Tayton (2007) describes the anteversion angle as ‘the anterior component of a 3D neck-shaft angle’. In his study, he suggests that the anteversion angle helps reducing the horizontal moment in the proximal femur in daily activity loading. It would thus make sense that a raised anteversion angle offered some protection against fracture during longitudinal compression. It is, however, not certain that the anteversion angle would impact fracture risk in side fall.

Despite its reduced neck-shaft angle, Femur 2a fractures at a significantly lower load than the mean femur and Femur 2b. The other main feature accounted for in PC2 is the shaft width, reduced in Femur 2a. It can thus be inferred from the present observations that a reduced shaft width significantly increases fracture risk, potentially via the resulting thinner shaft cortex, which has been correlated with increased fracture risk (Gregory and Aspden 2008; Partanen et al. 2001).

Although numerous studies report a correlation between a larger hip axis length (HAL) and an increased fracture risk (Boonen et al. 1995; Gnudi et al. 1999; Nakamura et al. 1994), there is no consensus in the literature regarding a relationship between larger femoral axis length (FAL) and increased fracture risk: some studies report a positive correlation (El-Kaissi et al. 2005; Center et al. 1998), others a negative correlation (Karlsson et al. 1996), and some no significant relationship (Calis et al. 2004; Yang et al. 1999). This lack of consensus might stem from the two-dimensional nature of the images often used to measure morphometric parameters, as FAL is likely to have components in three dimensions. In addition, many clinical studies do not differentiate between the origin of the fractures (common side fall or other loading scenario) which might prevent some trends from emerging. No real trend can be isolated in this study either, although the weakest bone presents the shortest FAL and the two strongest ones present FALs above that of the mean femur. It is interesting to note that the models with longer FAL also tended to be wider/thicker or have a higher neck-shaft angle. It is possible that bone width/thickness and neck-shaft angle (and therefore cortical thickness via adaptation) might compensate the increase in bending moment associated with a longer FAL in longitudinal loading. Future work involving testing of multiple PC combinations might help decouple the compensating features and isolate a clearer influence of FAL alone. Results regarding the relationship between larger femoral neck width and increased fracture risk reported in the literature are conflicting. Here again, some report positive correlation (Karlsson et al. 1996; Boonen et al. 1995; Alonso et al. 2000), negative correlation (Seeman et al. 2001; Karlamangla et al. 2004), or no significant influence (Ahlborg et al. 2005). The two strongest bones in this study present a relatively large neck width, which does not support a positive correlation with fracture risk; however, no definite trend can be isolated in this study.

From the results of this study and observations reported in the literature, it appears that a low neck-shaft angle, a slender neck, and long FAL geometry would be the worst combination (higher fracture risk) for longitudinal loading. It is interesting to see that this combination is not seen in the principal components of the statistical model, which is a reflection of the shapes seen in the population. In fact, the shape model shows variations where given one or two disadvantageous traits (e.g. slender, long FAL), there are one or two compensations (e.g. high neck-shaft angle). Overall, the results of this study help explain the shape model observations for the natural variation of femur shapes in a real population. These shapes tend to be mechanically stable for the longitudinal loading seen in regular gait.

### Limitations and future work

The models used in this study consider a set of common physical activities including walking, sit-to-stand, stand-to-sit, and going up and down the stairs. However, people engage in many other functional activities not considered here such as running, squatting, kneeling, child-bearing, sleeping on their side, or horse-riding. The frequency of these activities can vary with sex, culture, region, and history. Certain geometries might be more efficient for these other tasks at the expense of longitudinal mechanical stability.

A related limitation of this study is the use of the same set of loading conditions for the generation of all seven models. It is justified to study the impact of outer morphology on adaptation to a fixed loading scenario. However, it implies that the changes in moment arms of the muscles and hip contact forces generated by the changes in morphologies are not taken into consideration in the static optimisation step of the MSK simulations, although they could affect its results. It is indeed possible that subjects with different femoral morphologies would rely on different muscle recruitment strategies to perform the same task. Future work should thus include morphology-specific MSK modelling. Specifically, potential scaling laws for muscle and joint forces as femur shape PCs vary should be investigated.

Another limitation of this study is its small scale, with only three points along each main principal component being tested. The results allow for the tentative observation of some trends, and this study strongly suggests that external shape influences internal structure and fracture risk. However, a clearer and more comprehensive picture might be obtained with a higher number of data points, and more specifically with data points associated with combinations of variations along several PCs. It should be noted that the shape model used here was defined for male and female combined. Some of the six non-mean generated shapes would be more common in one sex than the other. Furthermore, it should also be noted that the femur chosen as the mean reference in this study does not exhibit mean PC1, as the femur length was kept consistent with the MSK model. PC1 refers mostly to the femur length, but also impacts other features such as neck-shaft angles. As a result, the mean reference femur in this study will exhibit small differences with the general population mean.

Finally, the outer shape of each femur generated was fixed in this study, while it is known shape as well as structure exhibits changes in response to mechanical stimuli (Bass et al. 2002; Anderson and Trinkhaus 1998). Further investigation should focus on the mechanical drivers involved in the development of the external shape of the bone, jointly with the internal structure adaptation.
